# Expeditious, mechanochemical synthesis of BODIPY dyes

**DOI:** 10.3762/bjoc.9.89

**Published:** 2013-04-23

**Authors:** Laramie P Jameson, Sergei V Dzyuba

**Affiliations:** 1 Department of Chemistry, Texas Christian University, Fort Worth, TX 76129, USA

**Keywords:** BODIPY, condensation, fluorescent dye, mechanochemistry, solvent-free

## Abstract

BODIPY dyes have been synthesized under solvent-free or essentially solvent-free conditions, within about 5 minutes in an open-to-air setup by using a pestle and mortar, with yields that are comparable to those obtained via traditional routes that typically require reaction times of several hours to days.

## Introduction

BODIPY dyes are fluorescent organic molecules, which have received a lot of attention in recent years due to their favorable chemical and physical characteristics, including high quantum yields and tunable fluorescent properties as well as high thermal and chemical stabilities [1–3]. As such, BODIPY and their derivatives have found widespread utility in a variety of different applications including biological [4–7] and light-harvesting systems [8–11].

**Scheme 1:**
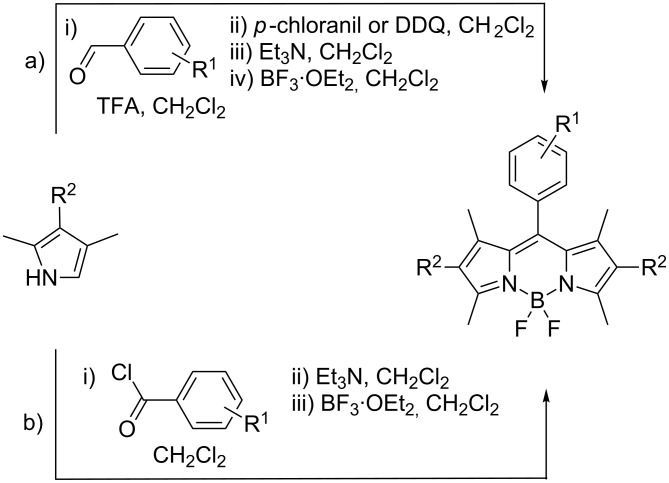
Literature preparations of symmetric, *meso*-substituted BODIPY dyes.

Despite wide applicability, the synthetic access to BODIPY dyes is neither facile nor efficient nor expeditious. The synthesis of BODIPY dyes is typically achieved by one of two different one-pot procedures (Scheme 1) [1–2]: (a) Lewis acid (usually trifluoroacetic acid, TFA) catalyzed condensation between a 2-substituted pyrrole and an aldehyde, followed by oxidation with DDQ or *p*-chloranil, and subsequent treatment with base (Et_3_N or Hunig’s base are typically used) and BF_3_·OEt_2_; (b) pyrrole condensation with an acid chloride and subsequent treatment with base (also usually Et_3_N or Hunig’s base as in procedure a) followed by treatment with BF_3_·OEt_2_.

While the synthesis of BODIPY dyes from the corresponding acid chloride (Scheme 1b) is frequently reported to give somewhat better yields (albeit reaction times of several days are often encountered), the structural diversity of commercially available acid chlorides is limited, and they must be prepared from the corresponding acid. Furthermore, the sensitivity of acid chlorides to moisture imposes additional constrains. Accordingly, the condensation of pyrroles with aldehydes (Scheme 1a) is the more commonly utilized synthetic approach [1–2]. Notably, both approaches are carried out under an inert atmosphere. Overall, based on literature accounts, BODIPY synthesis requires long reaction times (several hours to several days) and isolation of the dyes is performed by chromatography, with poor to moderate (10–50%) yields.

In this light, a more expedient synthesis of the BODIPY dyes is desired. Toward this end, we reasoned that the condensation step between the 2-substituted pyrroles and aldehydes (or acid chlorides) should be more facile at higher concentrations and probably most efficient under neat conditions. All the subsequent steps should also, in principle, benefit from high concentrations.

## Results and Discussion

**Scheme 2:**
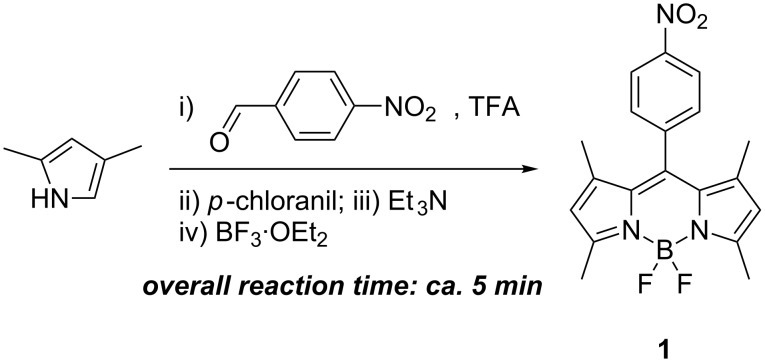
Expeditious synthesis of dye **1**.

As a trial reaction, we attempted a mechanochemical synthesis of BODIPY dye **1** [12] with commonly used reagents. The condensation of 2,4-dimethylpyrrole and 4-nitrobenzaldehyde was performed with grinding by using a simple mortar and pestle, followed by the addition of a few drops of TFA (complete consumption of the aldehyde was confirmed by TLC) and oxidation with *p*-chloranil. The reaction mixture was subsequently treated with Et_3_N and BF_3_·OEt_2_ to afford BODIPY dye **1** (Scheme 2). All reagents were added sequentially with grinding in ca. 5 minutes. Each step was accompanied by a color change. The desired product was isolated in 29% yield, which was comparable to those reported in literature protocols, 24–40% (Supporting Information File 1, Table S1), but in a substantially shorter reaction time. The reported reaction times for the synthesis of dye **1** range from 5 hours to ca. 12 hours (or overnight).

In view of this positive result (Table 1, entry 1), we attempted to optimize this procedure (Table 1). Specifically, several oxidizing agents were tried, including the typically used DDQ as well as Ce(NH_4_)_2_(NO_3_)_6_. However, *p*-chloranil afforded the highest isolated yield (Table 1, entries 1–3). We also found that adding small amounts of CH_2_Cl_2_ (1–2 mL) prior to the addition of *p*-chloranil allowed for a more efficient grinding to take place.

**Table 1:** Optimization of reaction conditions for the synthesis of dye **1**.

Entry	Oxidant	Base	Yield, %^a^

1	*p*-chloranil	Et_3_N	29
2	DDQ	Et_3_N	7
3	Ce(NH_4_)_2_(NO_3_)_6_	Et_3_N	0
4	Ce(NH_4_)_2_(NO_3_)_6_	DBU	8
5	*p*-chloranil	DBU	24^b^
6	*p*-chloranil	K_2_CO_3_	0
7	*p*-chloranil	NaOH	0

^a^Isolated yield, after column chromatography; ^b^yields varied from 8 to 24%.

Next, we examined several different bases for the deprotonation of the dipyrrolium species. Organic bases such as Et_3_N and DBU (Table 1, entries 1 and 5), proved to be most effective. Although DBU gave comparable results to Et_3_N, variations in yields were observed, likely due to the air-sensitive nature of DBU (Table 1, entry 5). Et_3_N, however, appeared to give reproducible results. In contrast, inorganic bases added as solids (K_2_CO_3_ and NaOH, Table 1, entries 6 and 7, respectively) failed to give the desired product. Overall, this essentially solvent-free, 5-minute procedure can be performed using the same reagents as the solution synthesis.

Finally, we also experimented with isolation procedures of the crude reaction mixture and found that diluting the mixture with CH_2_Cl_2_ and subsequently washing the organic layer with saturated Na_2_CO_3_ solution (to remove the hydroquinone byproduct), then brine, followed by column chromatography on silica gel gave satisfactory results. It is worth pointing out that isolation and purification of BODIPY dyes appeared to be problematic regardless of the synthetic procedure, i.e., under solution (literature accounts) or mechanochemical (current work) conditions. Vigorous shaking should be avoided during the extraction steps as it led to the formation of stable emulsions, which significantly lengthened the isolation process, and also decreased the overall efficiency of the reaction. However, we found that avoiding the extraction step altogether resulted in lower isolated yields of the BODIPY dyes. Also, CHCl_3_ was found to be a superior solvent for column chromatography as compared to CH_2_Cl_2_.

With the optimized conditions at hand, we prepared several BODIPY dyes by using commercially available aldehydes and either 2,4-dimethylpyrrole or 3-ethyl-2,4-dimethylpyrrole (Table 2) [13]. The reaction appeared to be insensitive to the electronic effects of the aldehyde, as both electron-donating and electron-withdrawing groups were equally tolerated. The yields were found to be reasonably comparable to those reported in literature (Supporting Information File 1, Table S1). Yet, the reaction times are drastically reduced from hours and several days (the latter holds true, for example, for dyes **4**, **6**, **7**) to just 5 minutes.

**Table 2:** 5-minute synthesis of BODIPY dyes.

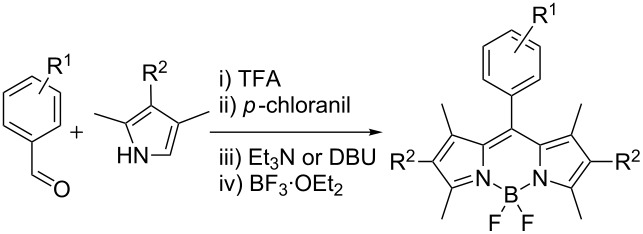			

Dye	R^1^	R^2^	Yield, %^a^

**1**	4-NO_2_	H	29
**2**	4-OCH_3_	H	15
**3**	4-CN	H	13
**4**	4-ethynyl	H	32
**5**	3-OH	Et	10^b^
**6**	4-CF_3_	Et	22
**7**	C_6_H_4_R^1^ = 4-pyridyl	Et	15

^a^Isolated yield, after column chromatography; ^b^23% yield when DBU was used as a base.

**Scheme 3:**
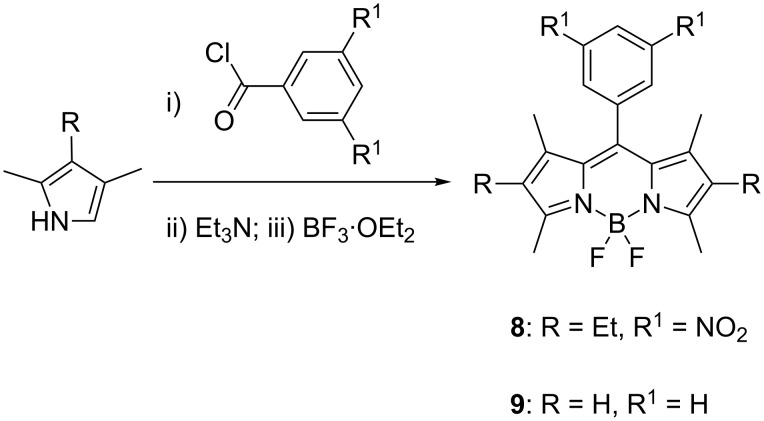
5-minute synthesis of dyes **8** and **9**.

Furthermore, we examined whether acid chlorides would be suitable substrates for this procedure (Scheme 3). It appeared that the condensation between pyrroles and acid chlorides produced dyes **8** and **9** in relatively low yields of 21% and 10%, respectively. It should be pointed out that in the case of dye **9** (and unlike the synthesis of **8**), the condensation step between 2,4-dimethylpyrrole and benzoyl chloride appeared to be sluggish. Specifically, with dye **8**, after 1 minute of grinding the pyrrole was consumed as judged by TLC. However, with dye **9**, a considerable amount of pyrrole remained (extending the grinding time to 5 minutes did not improve the efficiency of this step, as noted by TLC), which could have been the reason for the low yield of the dye **9**. Low yields might have been expected due to the significantly more moisture-sensitive nature of the acid chlorides as compared to the aldehydes. Conventional procedures, which utilize solvents and are performed under inert atmosphere for extended periods of time, afford BODIPY dyes in 30–50% yields [13–14]. Similar to the aldehyde-based synthesis of BODIPY dyes (Table 2), substituting Et_3_N with DBU led to inferior results: yields of 7% and 5% for dyes **8** and **9**, respectively, were obtained.

**Scheme 4:**
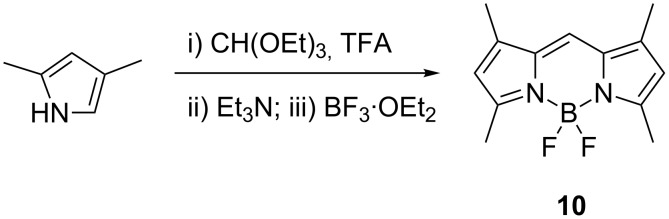
5-minute synthesis of dye **10**.

In addition, we explored the synthesis of BODIPY dye **10**, which does not have a substituent at the *meso*-position (Scheme 4), using triethyl orthoformate as the aldehyde component. Similar to all other cases, the literature synthesis of this BODIPY dye was performed in solution and required several hours [15]. The synthesis could be accomplished under solvent-free conditions within 5 minutes, using the same reagents, in 29% yield. An equimolar amount to TFA was used, in order to cleave the acetal to produce the aldehyde, which was condensed with the pyrrole.

## Conclusion

In conclusion, the developed procedure provides a rapid access to BODIPY dyes with reaction times being reduced from several hours or even days to only 5 minutes, while also eliminating large volumes of solvents. The syntheses of BODIPY dyes that used aldehydes as starting materials appeared to be superior to the approach that used acid chlorides. In addition, the unsubstituted BODIPY dye in the *meso*-position was also prepared via an acid-catalyzed triethyl orthoformate condensation with a pyrrole. Studies on improving the overall efficiency of this process, especially the isolation and purification of the BODIPY dyes, are in progress in our laboratory.

## Experimental

All reagents and solvents were from commercial sources (Sigma-Aldrich or Acros) and were used as received. Column chromatography was performed using silica gel (230–400 mesh) or basic alumina (Brockman I). Fraction collection was monitored by TLC (silica gel 60 F_254_) and the spots were visualized by UV. ^1^H NMR spectra were recorded on a Varian (300 MHz) spectrometer.

**General synthesis for the BODIPY dyes:** Aldehyde (2.5 mmol) and pyrrole (5.0 mmol) were mixed with a pestle and mortar. TFA (5 drops) was added via a pipette, while the mixture was ground with the pestle for about 30 seconds. To the resulting paste, CH_2_Cl_2_ or CHCl_3_ (2.0 mL) was added (to ensure an easier mixing of all components for the subsequent step; the presence/absence of CH_2_Cl_2_ or CHCl_3_ had no impact on the reaction time), followed immediately by the addition of *p*-chloranil (0.9 g, 3.7 mmol). The deep red/purple paste was ground for 1 minute, after which TEA (3.0 mL, 21.5 mmol) was added via a syringe. The resulting dark brown paste was ground with the pestle for 1 minute. Subsequently, BF_3_*·*OEt_2_ (3.0 mL, 23.7 mmol) was added slowly, dropwise, via a syringe and the mixture was ground for 1–2 minutes until a thick dark red paste was formed. *Caution: the addition of BF_3_·OEt_2_* *results in the formation of white fumes and some bubbling of the solution. Although we have not experienced any safety-related issues, this step (especially when done on a large scale) should be done behind a safety shield to avoid a potential splashing of the mixture.* The reaction mixture was dissolved in CH_2_Cl_2_ or CHCl_3_ (200 mL), transferred to a separation funnel, and washed with saturated Na_2_CO_3_ (3 × 200 mL) followed by brine (2 × 200 mL). The organic solvent was removed in vacuo, and the residue was subjected to column chromatography (silica gel, CHCl_3_) to give the desired product.

Further experimental details (including those for the synthesis of dye **10**) and spectroscopic characterization of all BODIPY dyes are given in Supporting Information File 1.

## Supporting Information

File 1 Experimental procedures and characterization data of prepared BODIPY dyes.
