# Supplementary material for: Expeditious, mechanochemical synthesis of BODIPY dyes
Source: Beilstein J Org Chem. 2013 Apr 23;9:786–90. doi: 10.3762/bjoc.9.89 (PMC3678757; doi:10.3762/bjoc.9.89)
Supplement: File 1 — Experimental procedures and characterization data of prepared BODIPY dyes. [file Beilstein_J_Org_Chem-09-786-s001.pdf]

Supporting Information

for

Expeditious, mechanochemical synthesis of BODIPY dyes

Laramie P. Jameson[1] and Sergei V. Dzyuba*[1§]

Address: [1]Department of Chemistry, Texas Christian University, Fort Worth, TX 76129, USA

Email: Sergei V. Dzyuba - s.dzyuba@tcu.edu

[§]Tel.: +1-817-257-6218; fax: +1-817-257-5851.

* Corresponding author

Experimental procedures and characterization data of prepared BODIPY dyes.

Table of Contents:

Materials and methods……...…………………………………………………. S2

Synthesis and spectroscopic characterization of BODIPY dyes……………… S2

Table S1...…………………………………………………………………….. S6

References……………………………………………………………………. S7

Materials and methods:

All reagents and solvents were from commercial sources (Sigma-Aldrich or Acros) and were used as received. Column chromatography was performed using silica gel (230–400 mesh) or basic alumina (Brockman I). Fraction collection was monitored by TLC (silica gel 60 F_{254}) and the spots were visualized by a hand-held UV lamp.
^{1}H NMR spectra were recorded on a Varian (300 MHz) spectrometer. ^{1}H NMR chemical shifts are reported in ppm on the δ-scale relative to tetramethylsilane (δ = 0.00). Multiplicities are reported as singlet (s), broad singlet (bs), doublet (d), triplet (t), quartet (q), multiplet (m).

Synthesis and spectroscopic characterization of BODIPY dyes

Dyes **1**–**6** were prepared according to the general procedure given in the main manuscript.

Dye 1: [1]

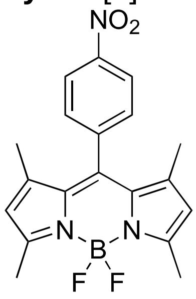

1

^{1}H NMR (300 MHz, $CDCl_3$) 8.39 (d, *J* = 8.7 Hz, 2H), 7.54 (d, *J* = 8.7 Hz, 2H), 6.02 (s, 2H), 2.57 (s, 6H), 1.37 (s, 6H).

Dye 2: [2]

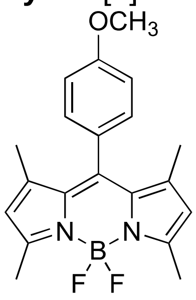

2

^{1}H NMR (300 MHz, $CDCl_3$) 7.15 (d, *J* = 9.0 Hz, 2H), 6.99 (d, *J* = 8.7 Hz, 2H), 5.96 (s, 2H), 3.85 (s, 3H), 2.54 (s, 6H), 1.42 (s, 6H).

Dye 3: [3]

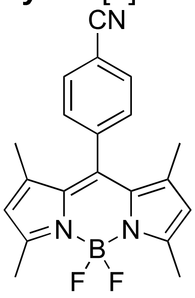

3

^{1}H NMR (300 MHz, $CDCl_3$) 7.82 (d, *J* = 7.6 Hz, 2H), 7.47 (d, *J* = 7.6 Hz, 2H), 6.01 (s, 2H), 2.56 (s, 6H), 1.35 (s, 6H).

Dye 4: [4]

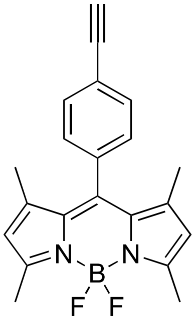

4

^{1}H NMR (300 MHz, $CDCl_3$) 7.62 (d, *J* = 7.8 Hz, 2H), 7.25 (d, *J* = 7.8 Hz, 2H), 5.98 (s, 2H), 3.19 (s, 1H), 2.55 (s, 6H), 1.39 (s, 6H).

Dye 5: [5]

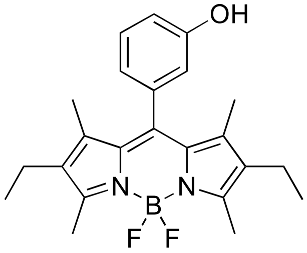

5

Synthesized according to the general procedure. Isolation and purification: The reaction mixture was dissolved in CH_2Cl_2 (200 mL), transferred to a separation funnel, and washed with 1 M HCl (200 mL) followed by brine (200 mL). The organic solvent was removed in vacuo and the residue subjected to column chromatography (silica gel, $CHCl_3$) to give the desired product.

^{1}H NMR (300 MHz, $CDCl_3$) 7.35 (t, *J* = 7.8 Hz, 1H), 6.94 (dd, *J* = 7.8, 2.4 Hz, 1H), 6.85 (d, *J* = 7.5 Hz, 1H), 6.77 (s, 1H), 5.09 (bs, 1H), 2.53 (s, 6H), 2.30 (q, *J* = 7.5 Hz, 4H), 1.37 (s, 6H), 0.98 (t, *J* = 7.5 Hz, 6H).

Dye 6: [6]

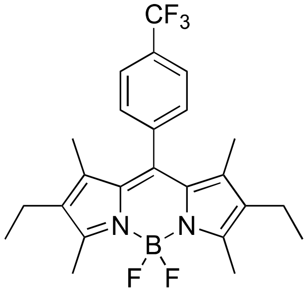

6

^{1}H NMR (300 MHz, $CDCl_3$) 7.77 (d, *J* = 7.5 Hz, 2H), 7.46 (d, *J* = 8.0 Hz, 2H), 2.54 (s, 6H), 2.30 (q, *J* = 7.5 Hz, 4H), 1.25 (s, 6H), 0.98 (t, *J* = 7.5 Hz, 6H).

Dye 7: [7]

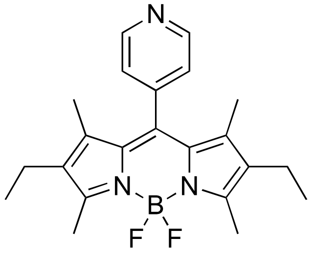

7

Prepared and isolated following the general procedure. Purification: subjected to column chromatography (basic Al_2O_3/Brockman I, $CHCl_3$) to give the desired product.

^{1}H NMR (300 MHz, $CDCl_3$) 8.77 (d, *J* = 5.7 Hz, 2H), 7.31 (d, *J* = 5.7 Hz, 2H), 2.54 (s, 6H), 2.30 (q, *J* = 7.6 Hz, 4H), 1.31 (s, 6H), 0.98 (t, *J* = 7.5 Hz, 6H).

BODIPY dyes **8** and **9** were prepared according to the general procedure for the BODIPY dyes, except that the corresponding acid chlorides were used instead of the aldehydes and TFA was omitted.

Dye 8: [8]

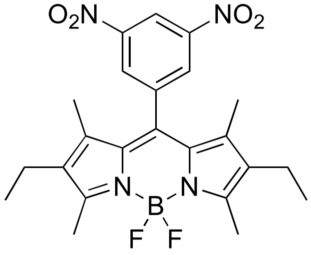

8

^{1}H NMR (300 MHz, $CDCl_3$) 9.19 (t, *J* = 2.1 Hz, 1H), 8.57 (d, *J* = 2.1 Hz, 2H), 2.56 (s, 6H), 2.31 (q, *J* = 7.7 Hz, 4H), 1.26 (s, 6H), 1.00 (t, *J* = 7.5 Hz, 6H).

Dye 9: [9]

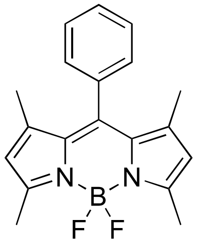

9

^{1}H NMR (300 MHz, $CDCl_3$) 7.48 (m, 3H), 7.28 (m, 2H), 5.98 (s, 2H), 2.56 (s, 6H), 1.37 (s, 6H).

Dye 10: [10]

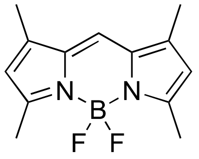

10

2,4-Dimethylpyrrole (0.19 mL, 1.8 mmol) was mixed with triethyl orthoformate (0.15 mL, 0.9 mmol) with a pestle and mortar. TFA (0.1 mL) was added via syringe to yield a red solid, which was ground for about 1 minute, after which TEA (1.5 mL, 10.8 mmol) was added via syringe. The resulting dark brown paste was ground with the pestle for 1 minute to give a yellow suspension. Subsequently, $BF_3{\cdot}OEt_2$ (1.5 mL, 11.9 mmol) was added slowly, dropwise via syringe and the mixture was ground for 1–2 minutes until a thick dark green mixture was formed. *Caution: the addition of $BF_3{\cdot}OEt_2$ results in the formation of white fumes and bubbling of the solution. Although we have not experienced any problems, this should be done behind a safety shield to avoid a potential splashing of the mixture.* The reaction mixture was dissolved in $CHCl_3$ (20 mL), transferred to a round-bottom flask, and the volatiles were removed in vacuo. The residue was subjected to column chromatography (silica gel, $CHCl_3$) to give the desired product as a dark red solid in 29% yield.

^{1}H NMR (300 MHz, $CDCl_3$) 7.04 (s, 1H), 6.05 (s, 2H), 2.53 (s, 6H), 2.25 (s, 6H).

Table S1: Reported synthesis of selected BODIPY dyes.

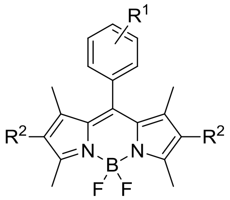

BODIPY	R^1	R^2	Reaction time[a]	Yield, % [ref]
**1**	4-NO_2	H	5 hours	30 [1a]
**1**	4-NO_2	H	12 hours & 50 min	40 [1b]
**1**	4-NO_2	H	12.5 hours	24 [1c]
**2**	4-OCH_3	H	16.5 hours	28 [2a]
**2**	4-OCH_3	H	18.5 hours	38 [2b]
**2**	4-OCH_3	H	5 hours & 10 min	NR[b] [2c]
**3**	4-CN	H	until aldehyde is consumed + 1 hour	15 [3a]
**4**	4-ethynyl	H	28 hours & 45 min	28 [4a]
**4**	4-ethynyl	H	18.5 hours	28 [4b]
**4**	4-ethynyl	H	46 hours & 30 min	8 [4c]
**4**	4-ethynyl	H	25 hours & 50 min	8 [4d]
**4**	4-ethynyl	H	14 hours & 10 min	30 [4e]
**4**	4-ethynyl	H	16 hours and 15 min	29 [4f]
**5**	3-OH	Et	until aldehyde is consumed + 5 min + identified time for the reaction with BF_3-OEt_2O	38 [5]
**6**	4-CF_3	Et	2 days & 2 hours & 5 min	34[c] [6]
**7**	$R^1C_6H_4$ = 4-pyridyl	Et	1 day & 20 hours	13 [7a]
**7**	$R^1C_6H_4$ = 4-pyridyl	Et	2 days & 4 hours	9 [7b]
**7**	$R^1C_6H_4$ = 4-pyridyl	Et	4 days & 3 hours	40 [7c]

[a]combined reaction time; it was assumed that when a reaction time for a given step was reported as "overnight" it was equal to 12 hours; [b]not Reported; [c]dye **6** was prepared in 84% yield by treating the dipyrrin HCl salt with $Et_3N/BF_3{\cdot}OEt_2$ [6a]. The dipyrrin HCl salt was prepared in 41% over 48 hours upon condensation of the pyrrole with the aldehyde [6b].

References

1. (a) H. Lu, S. S. Zhang, H. Z. Liu, Y. W. Wang, Z. Shen, C. G. Liu, X. Z. You, *J. Phys. Chem*. A, **2009**, *113*, 14081; (b) T. Matsumoto, Y. Urano, T. Shoda, H. Kojima, T. Nagano, *Org. Lett*., **2007**, *9*, 3375; (c) T. Ueno, Y. Urano, H. Kojima, T. Nagano, *J. Am. Chem. Soc.,* **2006**, *128*, 10640.
2. (a) G. Meng, S. Velayudham, A. Smith, R. Luck, H. Liu, *Macromolecules*, **2009**, *42*, 1995; (b) M. Baruah, W. Qin, C. Flors, J. Hofkens, R. A. L. Valee, D. Baljonne, M. Van der Auweraer, W. M. De Borggraeve, N. Boens, *J. Phys Chem. A*, **2006**, *110*, 5998; (c) L. Jiao, C. Yu, J. Li, Z. Wang, M. Wu, E. Hao, *J. Org. Chem*., **2009**, *74*, 7525.
3. Y. Chen, J. Jiang, *Acta Cryst.,* **2011**, *E67*, o908.
4. (a) K. T. Kim, B. H. Kim, *Chem. Commun.*, **2013**, *49*, 1717; (b) Z. Li, R. Bittman, *J. Org. Chem.,* **2007**, *72*, 8376; (c) M. Benstead, G. A. Rosser, A. Beeby, G. H. Mehl, R. W. Boyle, *Photochem. Photobiol. Sci.*, **2011**, *10*, 992; (d) Y. Teki, H. Tamekuni, K. Haruta, J. Takeuchi, Y. Miura, *J. Mater. Chem.,* **2008**, *18*, 381; (e) M. Kondo, S. Furukawa, K. Hirai, S. Kitagawa, *Angew. Chem., Int. Ed*., **2010**, *49*, 5327; (f) A. Hayek, F. Bolze, C. Bourgogne, P. L. Baldeck, P. Didier, Y. Arntz, Y. Mely, J.-F. Nicoud, *Inorg. Chem.,* **2009**, *48*, 9112
5. C. Duams-Verdes, F. Miomandre, E. Lépicler, O. Galandau, T. T. Vu, G. Clavier, R. Méallet-Renault, P. Audebert, *Eur. J. Org. Chem*., **2010**, 2525.
6. (a) S. M. Crawford, A. Thompson, *Org. Lett.,* **2010**, *12*, 1424; (b) A. A.-S. Ali, J. Cipot-Wechsler, S. M. Crawford, O. Selim, R. L. Stoddard, T. S. Cameron, A. Thompson, *Can. J. Chem.,* **2010**, *88*, 725.
7. (a) G. Ulrich, R. Ziessel, *J. Org. Chem.,* **2004**, *69*, 2070; (b) G. Ulrich, R. Ziessel, *Tetrahedron Lett*. **2004,** *45,* 1949-1953; (c) M. A. H. Alamiry, A. Harriman, L. J. Mallon, G. Ulrich, R. Ziessel, *Eur. J. Org. Chem*., **2008**, 2774.
8. R. Ziessel, L. Bonardi, P. Retailleau, G. Ulrich, *J. Org. Chem.,* **2006**, *71*, 3093.
9. (a) Y. Chen, J. Zhao, H. Guo, L. Xie, *J. Org. Chem.,* **2012**, *77*, 2192; (b) Y. Yue, *New J. Chem.*, **2011**, *35*, 61; (c) W. Wu, H. Guo, W. Wu, S. Ji, J. Zhao, *J. Org. Chem.,* **2011**, *76*, 7056.
10. M. Sekiya, K. Umezawa, A. Sato, D. Citterio, K. Suzuki, *Chem. Commun.,* **2009**, 3047.
